# Calcium metaborate induced thin walled carbon nanotube syntheses from CO_2_ by molten carbonate electrolysis

**DOI:** 10.1038/s41598-020-71644-0

**Published:** 2020-09-15

**Authors:** Xirui Wang, Xinye Liu, Gad Licht, Stuart Licht

**Affiliations:** Department of Chemistry, George Washington University, Washington, DC 20052 USA

**Keywords:** Climate change, Materials for energy and catalysis

## Abstract

An electrosynthesis is presented to transform the greenhouse gas CO_2_ into an unusually thin walled, smaller diameter morphology of Carbon Nanotubes (CNTs). The transformation occurs at high yield and coulombic efficiency of the 4-electron CO_2_ reduction in a molten carbonate electrolyte. The electrosynthesis is driven by an unexpected synergy between calcium and metaborate. In a pure molten lithium carbonate electrolyte, thicker walled CNTs (100–160 nm diameter) are synthesized during a 4 h CO_2_ electrolysis at 0.1 A cm^−2^. At this low current density, CO_2_ without pre-concentration is directly absorbed by the air (direct air capture) to renew and sustain the carbonate electrolyte. The addition of 2 wt% Li_2_O to the electrolyte produces thinner, highly uniform (50–80 nm diameter) walled CNTs, consisting of ~ 75 concentric, cylindrical graphene walls. The product is produced at high yield (the cathode product consists of > 98% CNTs). It had previously been demonstrated that the addition of 5–10 wt% lithium metaborate to the lithium carbonate electrolyte boron dopes the CNTs increasing their electrical conductivity tenfold, and that the addition of calcium carbonate to a molten lithium carbonate supports the electrosynthesis of thinner walled CNTs, but at low yield (only ~ 15% of the product are CNTs). Here it is shown that the same electrolysis conditions, but with the addition of 7.7 wt% calcium metaborate to lithium carbonate, produces unusually thin walled CNTs uniform (22–42 nm diameter) CNTs consisting of ~ 25 concentric, cylindrical graphene walls at a high yield of > 90% CNTs.

## Introduction

CO_2_ is the most prominent of the greenhouse gases, and due to greenhouse gas increase the planet is heating up. Atmospheric CO_2_ concentration, which had cycled at 235 ±  ~ 50 ppm for 400 millennia until 1,850, is currently at 416 ppm and rising at a rapid annual rate incurring global planetary climate disruptions and habitat loss^1–4^. CO_2_ was regarded as such a stable molecule that its transformation into a non-greenhouse gas posed a major challenge^5^. Conventional methodologies of carbon nanomaterial production have a high carbon footprint. For example, Chemical Vapor Deposition, CVD, is an energy intensive, expensive process to produce carbon nanomaterial associated with an unusually massive carbon footprint of up to 600 tonnes of CO_2_ emitted per tonne of carbon nanomaterial product^6^.

In 2009 and 2010 a novel sunlight driven methodology to split CO_2_ into carbon and oxygen by molten carbonate electrolysis was introduced^7,8^. This process does not require sunlight, and it was demonstrated that using a molten carbonate and a variety of electrolytic configurations, the carbon product can be made into pure carbon nanomaterials, such as Carbon Nanotubes (CNTs)^9–24^. For example, this novel chemistry transforms the greenhouse Carbon dioxide to Carbon NanoTubes (C2CNT), and directly captures and CO_2_ from the atmosphere or from concentrated anthropogenic CO_2_ sources such as power plant exhaust. Several different carbon allotropes can be produced by C2CNT. This report focuses on the synthesis of thin walled CNTs from CO_2_ by a modification of this methodology.

The C2CNT process, has quantified the high affinity of molten carbonates to absorb both atmospheric and flue gas levels of CO_._, Utilizing the ^13^C isotope of CO_2_ to track in-flux, we have previously demonstrated in molten lithium carbonate that CO_2_ originating from the gas phase serves as the renewable carbon building blocks in the observed CNT product^10,11^. The net reaction is in accord with:

1 $${\text{Dissolution}}:\quad {\text{CO}}_{{2}} \left( {{\text{gas}}} \right) \, + {\text{ Li}}_{{2}} {\text{O}}\left( {{\text{soluble}}} \right) \to {\text{Li}}_{{2}} {\text{CO}}_{{3}} \left( {{\text{molten}}} \right)$$

2 $${\text{Electrolysis}}:\quad {\text{Li}}_{{2}} {\text{CO}}_{{3}} \left( {{\text{molten}}} \right) \to {\text{C}}\left( {{\text{CNT}}} \right)a + {\text{Li}}_{{2}} {\text{O }}\left( {{\text{soluble}}} \right) + {\text{O}}_{{2}} \left( {{\text{gas}}} \right)$$

3 $${\text{Net}}:\quad {\text{CO}}_{{2}} \left( {{\text{gas}}} \right) \to {\text{C}}\left( {{\text{CNT}}} \right) + {\text{O}}_{{2}} \left( {{\text{gas}}} \right)$$

**Figure 1:**
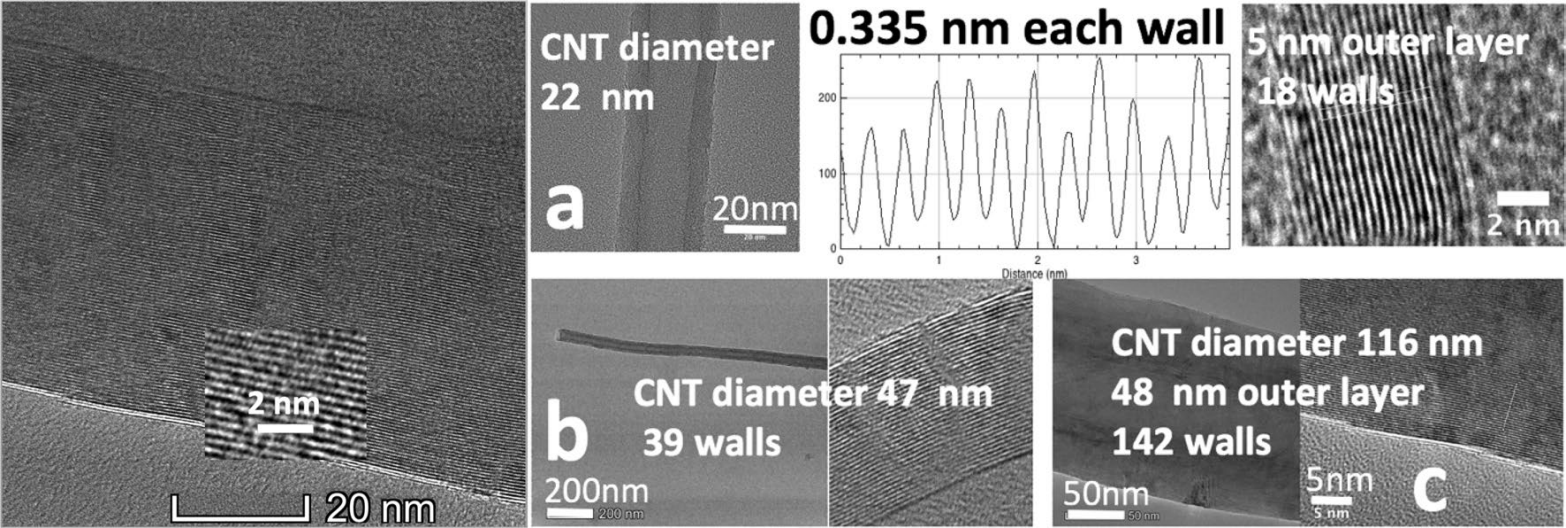
TEM of carbon nanotube walls in molten carbonated synthesized CNTs. The synthesis is by 0.2 (add space) A cm^2^ electrolysis in 770 °C Li_2_CO_33_ at a 5 cm^2^ coiled copper wire and Ni powder). Left: an expanded view of the CNT product after 90 min synthesis. The synthesis produces a pure CNT product whose diameter, and number of cylindrical graphene walls increases with electrolysis time. TEM of the synthesis product after **a**: 15, **b**: 30 or **c**: 90 min electrolysis.

**Figure 2:**
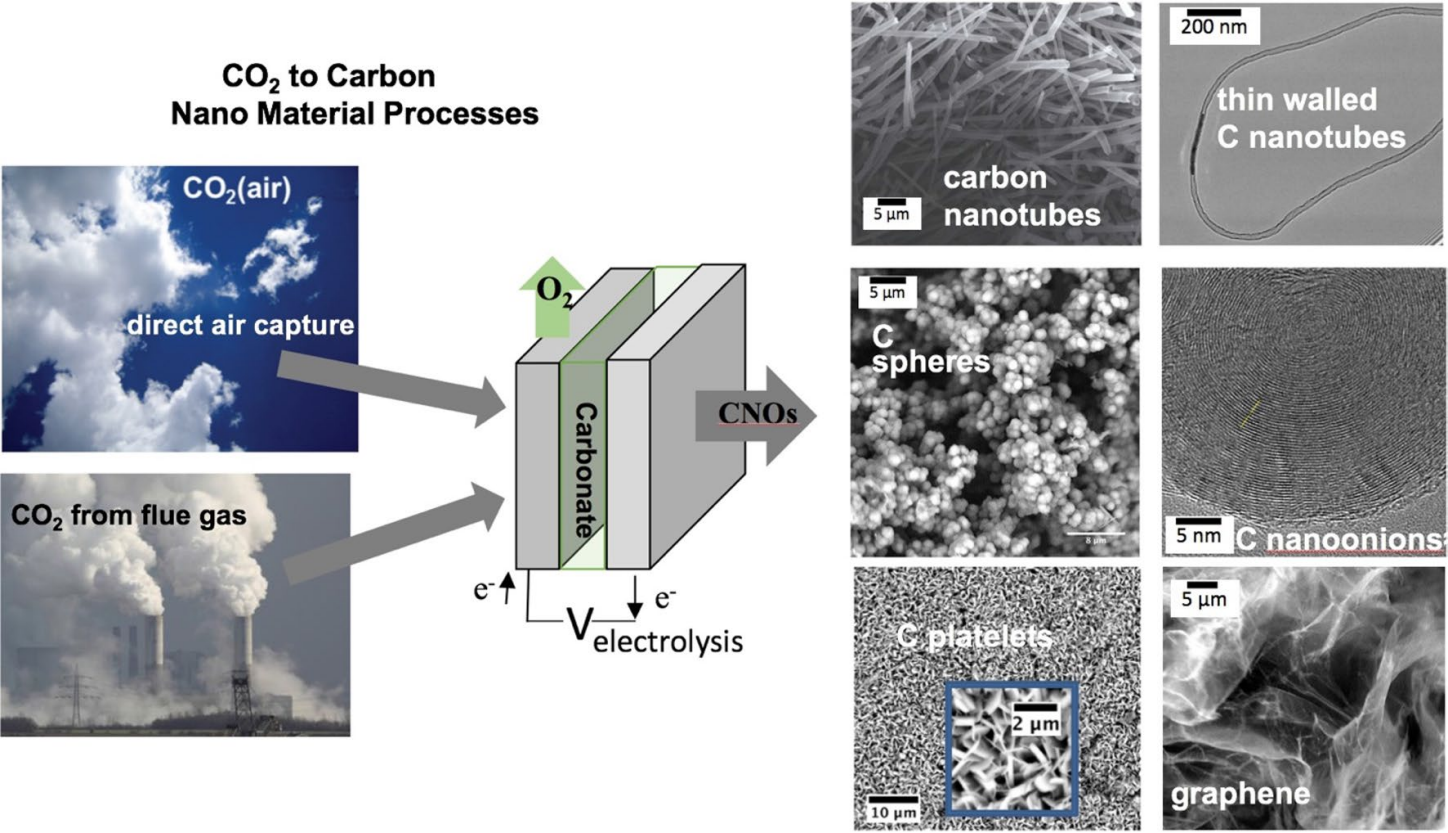
High yield electrolytic synthesis of carbon nanomaterials from CO_2_, either directly from the air or from smokestack CO_2_, in molten carbonate^11,19,22,23^.

An important component of the C2CNT growth process is transition metal nucleated growth, such as the addition of nickel powder which leads to clearly observable CNT walls as shown in Fig. 1. However, when these nucleation additives are purposely excluded during the synthesis, then the high yield synthesis of carbon nano-onions (spheres) (as shown in Fig. 2) or graphene are accomplished^19,22^.

Many different carbon allotropes can be produced by molten CO_2_ splitting also referred to as the Genesis Device™. The wide range of carbon nanomaterial morphologies observed shows the potential for tuning the product for uses in many different useful products. Here, we present synthesis of unusually thin walled CNTs, which would have particular use in applications requiring less rigid CNTs; for example, for nano connections within circuits, and conductive pastes^25^. Unlike previous methodologies to synthesize thin walled CNTs, this new methodology is inexpensive (using inexpensive materials and electrosynthesis) and is the only methodology which is carbon negative to help mitigate climate change. The synthesis finds an unexpected synergy by combining in a single step, a previous low yield but thin walled CNT electrosynthesis, and a boron doping CNT electrosynthesis. The new high yield product is significant both due to its morphology, and green carbon footprint as the methodology uses CO_2_ as a reactant making it carbon negative.

## Experimental

### Experiment materials and procedure

Lithium carbonate (Li_2_CO_3_, 99.5%), lithium oxide (Li_2_O, 99.5%), calcium oxide (CaO, 99.5%) and boric acid (H_3_BO_3_, 99.9%) are used as the electrolyte in this study.

### Electrolysis and purification

The electrolyte is pre-mixed in the noted ratios. Unlike early studies, such as shown in Fig. 1, that used 1 cm separated, horizontally aligned anodes and cathodes disks comprised of coiled wires, this study uses electrodes that are sheet metal and vertically immersed into the molten salt electrolyte. 0.25-inch-thick Muntz brass sheet is used as the cathode, and 0.04-inch-thick Nichrome (chromel A) sheet is used as the anode. The cathode is aligned (sandwiched) between two series connected anodes, and the cathode is spaced 1 cm from each of the anodes. The electrolyte and electrodes are contained in a rectangular stainless steel 304 case. Unlike, the experiments described in Fig. 1 which was at a constant current of 0.2 A/cm^2^ for different short intervals of time (15, 30 or 90 min), here, for the vertically immersed planar electrodes a constant current of 0.1 A/cm^2^ is applied for a constant time of 4 h. The electrolysis temperature is 770 °C. The raw product is collected from the brass cathode after the experiment and cooled down, followed by an aqueous wash procedure. The washed carbon product is separated by vacuum filtration. The washed carbon product is dried overnight at 60 °C in an oven yielding a black power product.

The coulombic efficiency of electrolysis is the percent of applied, constant current charge that was converted to carbon determined as:

4 $${1}00\% \times {\text{C}}_{{{\text{experimental}}}} {\text{/C}}_{{{\text{theoretical}}}}$$

This is measured by the mass of washed carbon product removed from the cathode, C_experimental_, and calculated from the theoretical mass, C_theoretical_ = (Q/nF) × (12.01 g C mol^−1^) which is determined from Q, the time integrated charged passed during the electrolysis, F, the Faraday (96,485 As mol^−1^ e^-^), and the n = 4 e- mol^−1^ reduction of tetravalent carbon.

### Characterization

Samples are analyzed by PHENOM Pro Pro-X SEM, FEI Teneo LV SEM, and by FEI Teneo Talos F200X TEM.

## Results and discussion

The electrolytic splitting of CO_2_ in molten carbonate electrodes can be conducted with a wide range of cathode materials including iron, steels, nickel, nickel alloys, Monel, copper and brass. The diameter of the CNTs grown on copper or on brass cathodes tends to be similar. In Fig. 1, concentric CNT walls separated by 0.335 nm, which is typical of the distinctive one atom thick separation of multiple graphene layers are observed. Figure 1 demonstrates when the electrolyte is conducted in pure Li_2_CO_3_ an increase in CNT diameter from 22 to 116 nm occurs when the constant current electrolysis time is increased from 15 to 90 min. The CNT is composed of concentric, cylindrical graphene walls spaced 0.335 nm apart. Alongside the increased diameter is an increase in the number of concentric CNT walls on each of the inner sides of the nanotube increase from 18 to 142 graphene layers. In pure Li_2_CO_3_, for 4 h, rather than 1.5 h electrosynthesis, the CNT continues to grow, and on the average the CNT diameter ranges from 100 to 160 nm, for example with repeat a 4 h constant current electrolyses.

**Figure 3:**
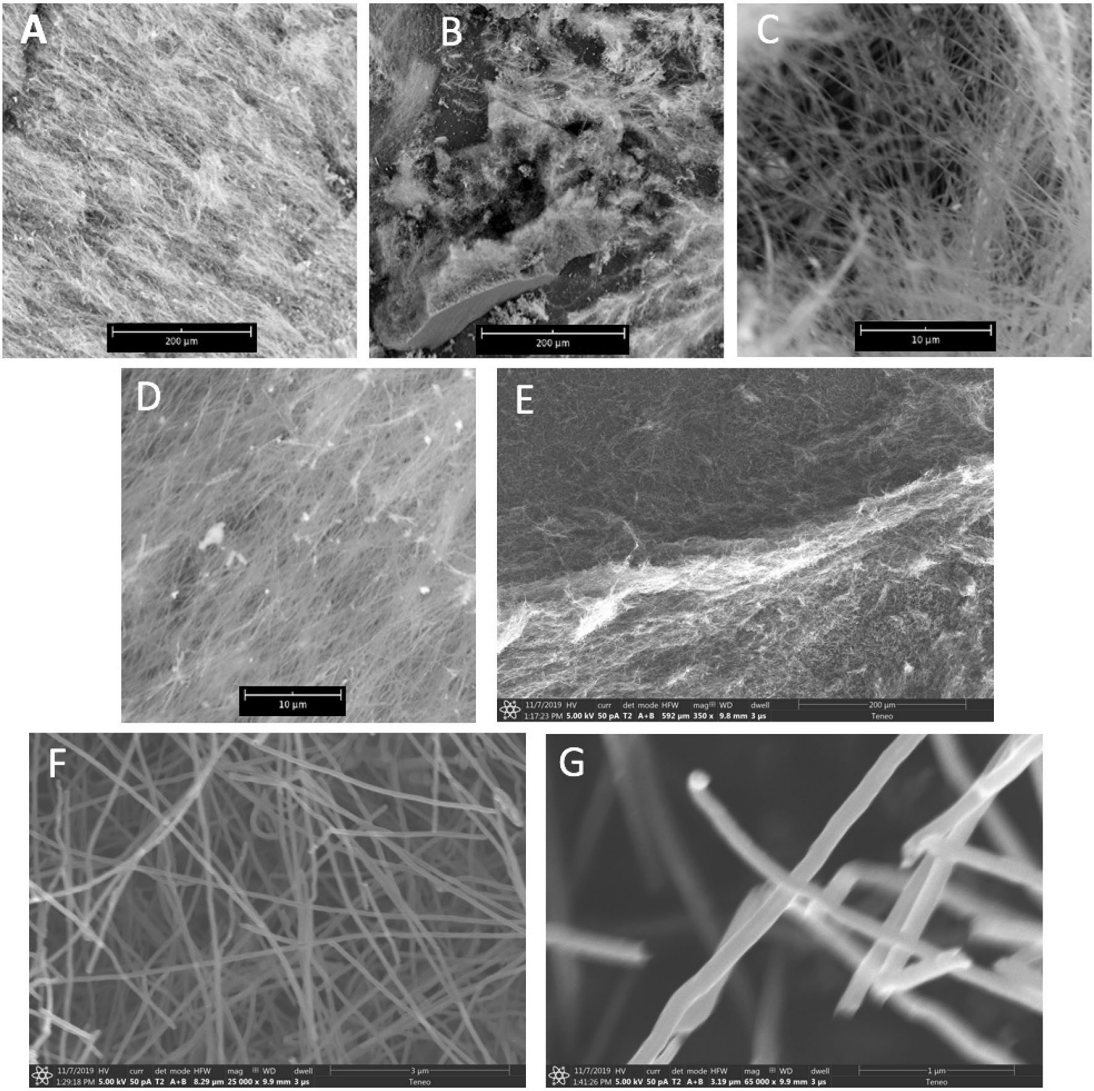
SEM (panels **A**–**G**) of the CNTs product of CO_2_ splitting in a molten Li_2_CO_3_ electrolyte containing 0.67 molal (2 wt%) Li_2_O. The scale bars in SEM (**A**–**E**) are respectively at 200, 200, 10, and 10 µm. The scale bars in TEM (**E**–**G**) are respectively at 200, 3 and 1 µm.

The electrolyte composition can affect the CNT diameter. Figure 3 presents SEM of the thinnest 4 h grown CNTs that had been observed. This is accomplished by addition of low concentrations of lithium oxide to the electrolyte. The CNTs are electrosynthesized in 770 °C Li_2_CO_3_ with 2 wt% Li_2_O electrolyte (0.67 mol of Li_2_O per kg Li_2_CO_3_) using a nickel alloy anode and brass cathode. At the relatively low current density of 0.1 A/cm^2^ applied (aluminum smelting by electrolysis of aluminum oxide typically occurs at 0.5–0.6 A/cm^2^), CO_2_ from the air (direct air capture) is sufficient to renew the electrolyte in accord with Eq. (3) and maintain the electrolyte level in accord with Eq. (1), and concentrated addition of CO_2_ is not required and not added. Nickel chromium alloy anodes and brass cathodes have been shown to be particularly stable for repeated use in CO_2_ splitting by molten carbonate electrolysis^21^. After the synthesis, the extracted cathode is cooled and the solid product readily is peeled off the cathode and washed to remove excess electrolyte prior to microscopy. Panel B of Fig. 3 is of interest as it constitutes SEM of a product removed from the rear side (not facing the anode) of the cathode. In particular, a piece of the multilayer graphene sheet which first forms on the cathode, and from which the CNTs growth is evident in a manner consistent with the tip growth mechanism presented in reference 3. The product is ~ 98% uniform CNTs as determined by visual inspection of multiple SEMs and TEM. Repeat experiments using the 2 wt% Li_2_O in Li_2_CO_3_ electrolyte, the coulombic efficiency was consistently 97% to 100%. Lower concentrations of lithium oxide resulted in thicker diameter and CNTs, and greater than 2 wt% added lithium oxide did not further decrease the observed CNT product thickness. The diameter of representative samples of the CNTs was measured with the nano-caliper function of the Phenom SEM and varied from ~ 50 to 80 nm, or approximately half the diameter of CNTs electrosythesized in pure Li_2_CO_3_ without added Li_2_O.

It had previously been demonstrated that the addition of 5–10 wt% LiBO_2_ to a Li_2_CO_3_ electrolyte, used in CO_2_ electrolysis, boron dopes the CNTs increasing their electrical conductivity tenfold^18^. It had also been shown the addition of alkali earth metal carbonates to a lithium electrolyte have a substantial effect on the carbon nanomaterial electrolysis product. For example, the addition of magnesium carbonate prevented the formation of CNTs, and the addition of CaCO_3_ inhibited, and diminished, but allowed the formation of CNTs resulting in a yield of only ~ 15 CNT product^15^. Interestingly, it was observed that those CNTs which did form in the Ca/Li mixed carbonate electrolyte had much thinner walls than those synthesized in pure lithium carbonate^15^.

Of the metaborate salts, and their molten phase counterparts, sodium metaborate is that which is most studied, which is likely due to its use in certain formulations of glass^26–30^. To a lesser extent calcium borate, CaB_2_O_4_ or CaO·B_2_O_3_, has also been studied^31–33^. Note that the boron in calcium metaborate has a ratio Ca to B to O ratio of 1:2:4, whereas the ratio in calcium borate, common name Gersely borate, Ca_3_(BO_3_)_2_ is 1:2/3:2.

Calcium metaborate in this study, was synthesized by the addition of calcium oxide and boric acid:

5 $${\text{CaO}} + {\text{2H}}_{{3}} {\text{BO}}_{{3}} \to {\text{CaO}} \cdot {\text{B}}_{{2}} {\text{O}}_{{3}} + {\text{3H}}_{{2}} {\text{O}}$$

**Figure 4:**
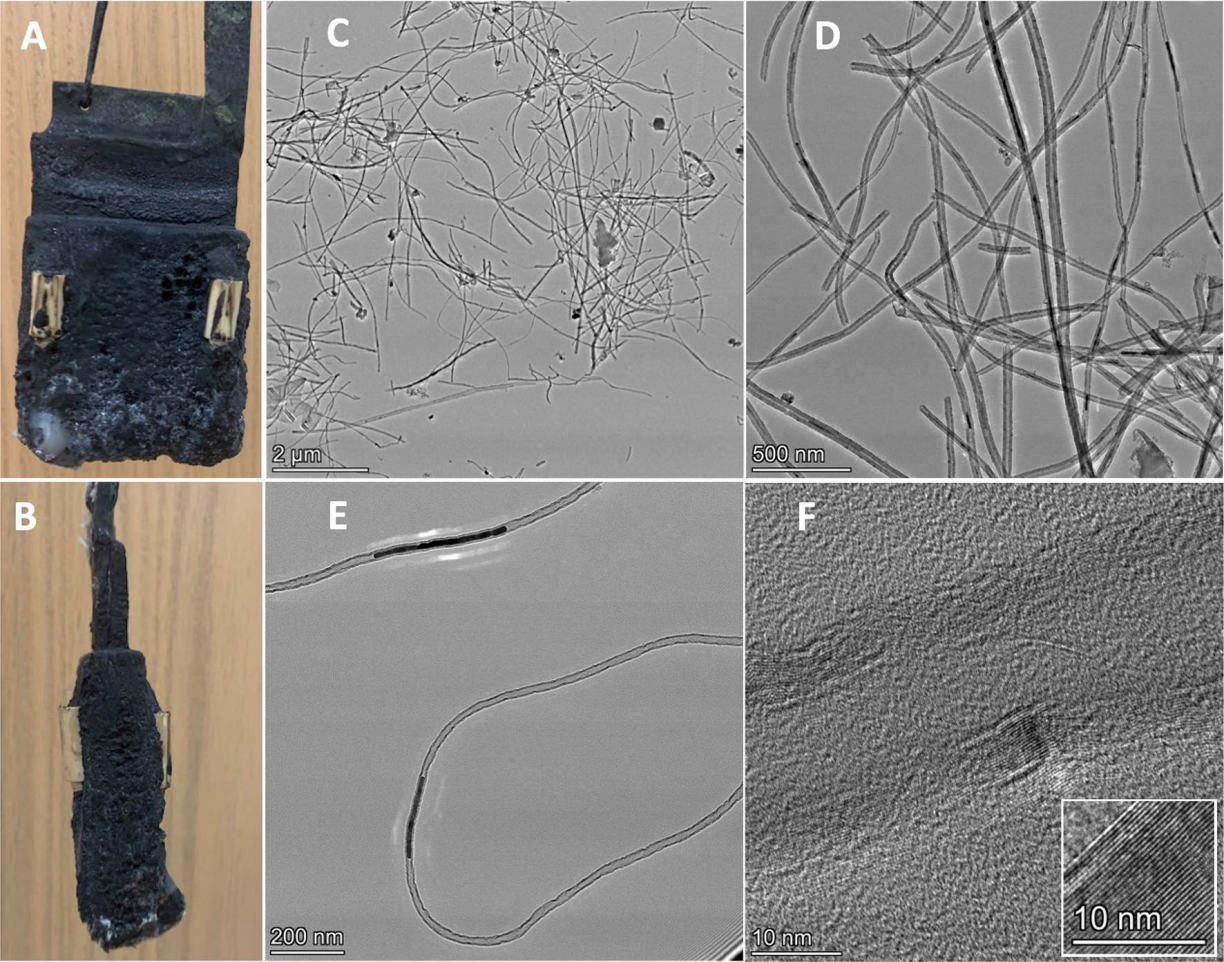
Photos of the extracted cathode (**A**,**B**) and TEM (of the CNT product) after CO_2_ electrolytic splitting in a molten Li carbonate electrolyte containing 0.67 molal calcium metaborate. The scale bars in the TEM are respectively at 2 µm (**C**), 500 nm (**D**), 200 nm (**E**) and 10 nm (**F**).

Specifically, 0.2 mol of Ca and 0.4 mol of boric acid were added to 300 g Li_2_CO_3_ and heated at 770 °C overnight to release all water as steam. The electrolysis of CO_2_ uses a molten electrolyte mix of 0.67 molal (7.7 wt%) CaO·B_2_O_3_ in Li_2_CO_3_, with a 6 cm by 7 cm brass cathode sandwiched between nichrome anodes. The electrolysis approaches 100% coulombic efficiency as measured according to Eq. (4), and the product consists of 2–6 µm length CNTs, and is marginally less pure (90% yield of CNTs) than the 0.67 molal Li_2_O synthesis. The cathode is extracted and cooled, after a 4 h electrolysis is shown on the left side of Fig. 4. White cylinders in the photo are alumina placed on the cathode to prevent shorting with the anode. The graphitization of the thin walls is demonstrated in the inset of panel F by the individual carbon nanotubes which are flat and separated by 0.34 nm, which is the same as graphene layers in graphite^11^.

**Figure 5:**
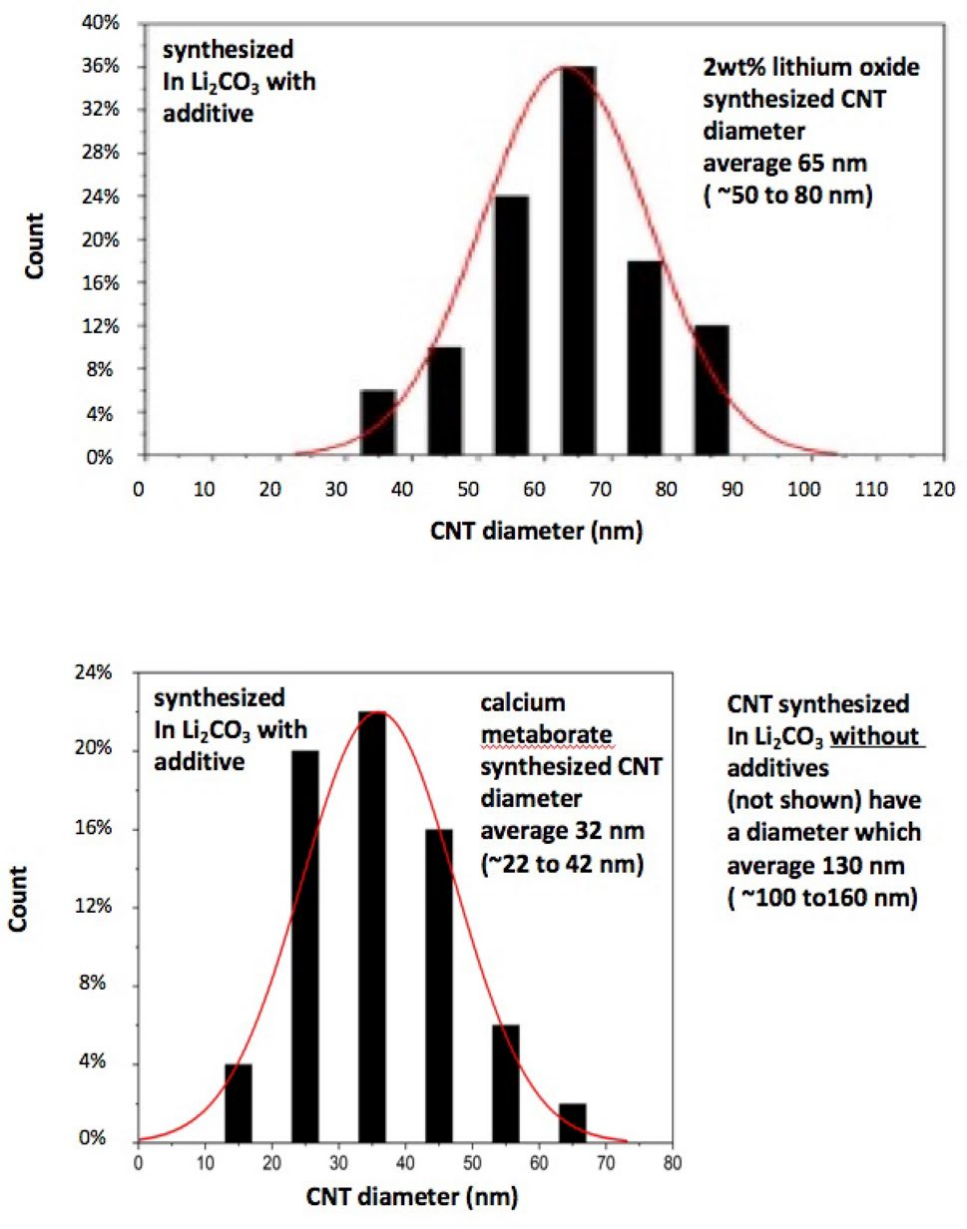
The distribution of CNT diameter size by count is compared between the CNT product formed om lithium carbonate electrolyte, either containing 0.67 m Li_2_O (top), or 0.67 m CaO·B_2_O_3_ (bottom). After the same electrolysis, but in a pure Li_2_CO_3_ electrolyte (without additives), the average CNT diameter after a 4 h electrolysis is ~ 130 to 160 nm.

The diameter of representative CNTs of the calcium borate in Li_2_CO_3_ electrosynthesized CNTs varied from ~ 22 to 42 nm which is considerably smaller than in similar pure lithium carbonate or lithium carbonate with lithium oxide electrolytes. The distribution of CNT diameter size by count is compared in Fig. 5 between the CNT product formed from Li_2_CO_3_ electrolyte, either containing 0.67 m Li_2_O (top), or 0.67 m CaO·B_2_O_3_ (bottom). The average CNT diameter after a 4 h electrolysis is 130 nm in Li_2_CO_3_ without additives, 65 nm with 0.67 m Li_2_O and 32 nm with 0.67 m CaO.B_2_O_3_.

## Conclusions

We present a new high yield pathway to produce thin walled carbon nanotubes. The process uses a calcium metaborate dissolved in a molten Li_2_CO_3_ electrolyte, and splits and consumes CO_2_ as the carbon source building blocks of the carbon nanotubes. Using equivalent 4 h CO_2_ 770 °C electrolyses at 0.1 A cm^−2^, the carbon CNT products of electrolyses in a pure molten lithium carbonate electrolyte, have a diameter of 100 to 160 nm. Those conducted in lithium carbonate containing 0.67 m Li_2_O have a diameter of 50 to 80 nm, while those containing 0.67 m CaO.B_2_O_3_ have a diameter of 22–42 nm diameter. Each range of carbon nanotube morphology has various applications in energy storage, high strength composites, conductive pastes and as catalysts.

In accord with Fig. 1, it is likely that the diameter may be decreased approximately eightfold by a 15 min, rather than 4 h electrolysis. In a similar manner, electrolyses conducted for the same period, but with a lower current density will likely also exhibit fewer CNT walls and smaller CNT diameters. The high yield, high coulombic efficiency molten carbonate electrosynthesis of single and double walled CNTs may be within reach and achieved by combining appropriate electrolyte additives, such as calcium metaborate, with low current density and short electrolysis times.
